# Syndrome de poland: à propos d’un cas et revue de la litterature

**DOI:** 10.11604/pamj.2017.26.12.11222

**Published:** 2017-01-05

**Authors:** Meriam Benzalim, Laila Berghalout, Sophia Elfakir, Hicham Jalal

**Affiliations:** 1 Service de Radiologie Pôle Mère-enfant, CHU Mohammed VI, Marrakech, Université Cadi Ayyad

**Keywords:** Syndrome Poland, imagerie, classification, Poland syndrome, imaging, classification

## Abstract

Le syndrome de Poland est une malformation congénitale rare associant à des degrés divers des anomalies thoraciques et du membre supérieur homolatéral. Nous rapportons le cas d'une fillette de 7 ans, explorée pour dépression de l'hémithorax gauche avec masse sous claviculaire homolatérale. La tomodensitométrie a montré que la déformation de la paroi thoracique était liée à l'absence des chefs à insertion sterno-costale du muscle pectoralis major gauche avec agénésie du pectoralis minor et hypoplasie des arcs antérieurs des six premières côtes correspondantes. Ces anomalies étaient rapportées à un syndrome de Poland. Un bilan général fait d'échographie abdominale, radiographies des deux mains, a été réalisé chez la patiente n'ayant révélé aucune malformation associée. Le syndrome de Poland résulte d'un défaut d'approvisionnement sanguin des éléments musculosquelettiques de la paroi thoracique pendant la vie fœtale. Il existe de nombreuses variantes du syndrome de Poland qui peuvent être mieux détectées par la TDM qui doit être réalisée chaque fois qu'il est disponible, sans omettre le rôle de la radiologie général dans la détection des malformations associées. La caractéristique de ce syndrome est l'agénésie des faisceaux sternocostaux du muscle pectoralis major. Son étiologie reste inconnue et discutée. Une anomalie vasculaire en serait la cause, sans que le primum movens de cette anomalie vasculaire soit connu.

## Abstract (en)

Poland syndrome is a rare congenital malformation associated with various degrees of thoracic and homolateral upper limb abnormalities. We report the case of a 7-year old girl who underwent exploration for depression of the left hemithorax associated with homolateral subclavicular mass. CT scan showed that the deformation of the thoracic wall was related to the absence of left pectoralis major muscle sterno-costal heads insertion associated with agenesis of the pectoralis minor muscle and hypoplasia of the anterior arches of the first six corresponding ribs. These abnormalities were related to Poland syndrome. General assessment was based on abdominal ultrasound, X-rays of both hands and did not show any associated malformation. Poland syndrome results from a deficit of blood flow to musculoskeletal elements of the chest wall during fetal life. There are many variants of Poland syndrome that can be best detected by CT scan and which must be performed whenever necessary, without omitting the role of general radiology in the detection of associated malformations. This syndrome is characterized by the agenesis of the sternocostal bundles of the pectoralis major muscle. Its etiology remains unknown and discussed. It can be caused by a vascular anomaly, without the primum movens of this vascular anomaly being known.

## Introduction

Le syndrome de Poland se caractérise par une agénésie du muscle pectoralis major et une agénésie mammaire associée ou pas à des anomalies du membre supérieur homolatéral [1–3]. La forme complète associe une agénésie des faisceaux sternocostaux du pectoralis major à une symbrachydactylie de la main homolatérale. Cet ensemble malformatif doit son nom à Alfred Poland, étudiant en anatomie, qui fut le premier à en donner une description complète en 1841 [4, 5]. Les anomalies thoraciques sont musculaires, ostéocartilagineuses et cutanéoglandulaires [5]. Dans de rares cas, gravement touchés, des anomalies des organes internes tels que les poumons, reins et cœur peuvent être associées [1, 3, 4]. Son incidence a été estimée à 1 à 3 par 100.000 nouveau-nés avec nette prédominance masculine [1, 5]. Le côté droit est affecté dans 75% des cas [1]. La cause du syndrome de Poland reste inconnue [1, 5, 6]. L'hypothèse d'interruption du flux sanguin au cours du développement fœtal pourrait expliquer l'agénésie du muscle pectoralis major [1, 4, 5].

## Patient et observation

**Figure 1:**
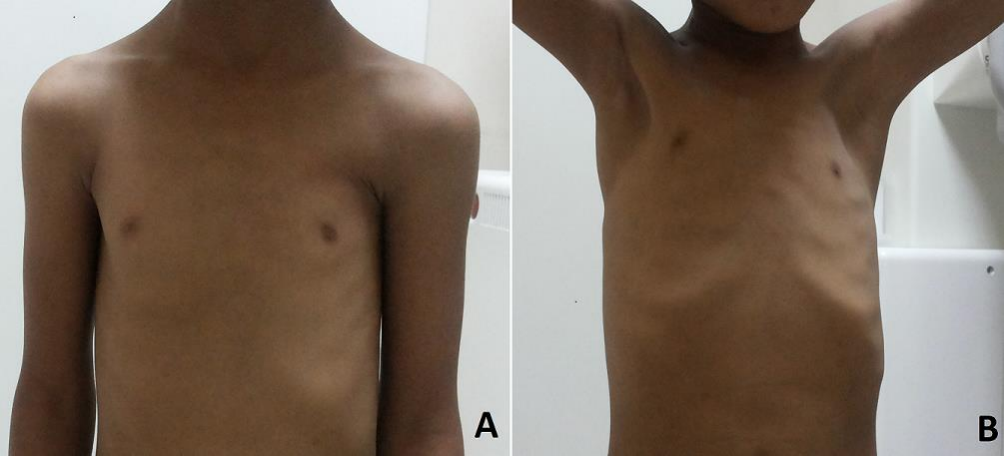
Aspect clinique de la patiente atteinte du syndrome de Poland; A) absence du muscle pectoralis major gauche; B) dépression de la région pectorale gauche

**Figure 2:**
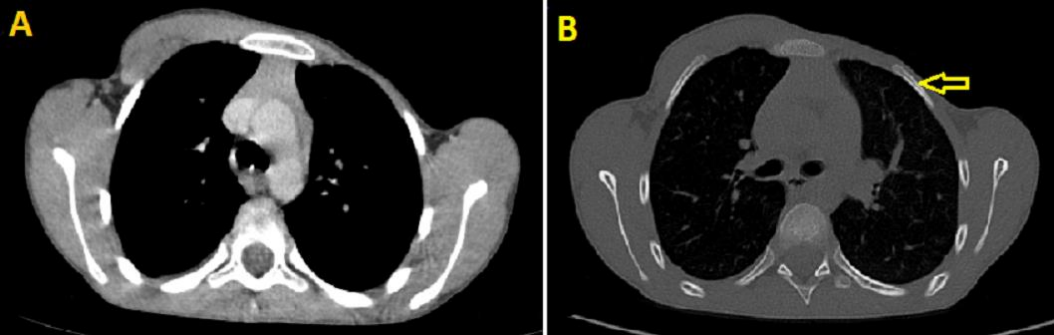
Images tomodensitométriques axiales du thorax en fenêtre médiastinale (A) et osseuse; (B) montrant; A) hypoplasie du muscle pectoralis major gauche avec absence des chefs à insertion sterno-costale; B) discret raccourcissement des cotes gauches en comparaison avec le coté controlatéral

Nous rapportons le cas d'une fillette de 7ans, née à terme par voie basse d'une grossesse mal suivie, sans notion de consanguinité ni de prise médicamenteuse au cours de la gestation. La constatation par la maman d'une déformation thoracique à type de dépression sous mammaire gauche associée à une masse sous claviculaire homolatérale mobile, a motivé la consultation d'un pédiatre. L'examen physique a mis en évidence la dépression de la paroi thoracique gauche responsable d'une asymétrie des deux hémithorax (Figure 1). L'examen neurologique et du squelette était normal. Une TDM thoracique a été réalisée objectivant une agénésie de la portion à insertion sterno-costale du pectoralis major gauche avec agénésie totale du pectoralis minor et hypoplasie des arcs antérieurs des six premières côtes homolatérales (Figure 2) correspondant à un syndrome de Poland. Un bilan malformatif fait d'échographie abdominale et de radiographies des deux mains a été réalisée, n'objectivant aucune malformation associée notamment rénale et des os des membres supérieurs.

## Discussion

Le syndrome de Poland est une anomalie congénitale rare impliquant des malformations musculosquelettiques de la paroi thoracique et du membre supérieur homolatéral [1–3]. Il a été décrit la première fois par Sir Alfred Poland en 1841 [7]. Il survient habituellement de façon sporadique, seuls quelques cas familiaux ont été rapportés [1, 7]. Il est d'étiologie inconnue. De nombreuses théories ont été proposées, mais il semble que l'interruption de l'approvisionnement en sang embryonnaire à l'artère sous-clavière, artères vertébrales et leurs branches, explique l'apparition du syndrome de Poland et ses variantes [7, 8]. Cette condition a généralement lieu au cours de la sixième semaine de gestation [7]. Ces perturbations vasculaires peuvent aussi expliquer l'apparition d'autres syndromes tels que le Möbius, Klippel-Feil et Adams-Oliver [7]. D'autres études ont incriminé l'exposition aux alcaloïdes de l'ergot au cours du premier trimestre de grossesse dans l'apparition du syndrome [7]. Depuis lors, de nombreuses variantes et anomalies associées à ce syndrome ont été décrites [9]. Ceux-ci incluent l'absence du pectoralis minor ou l'absence totale des deux muscles pectoraux, l'hypoplasie des côtes, hypoplasie de l'omoplate et de ses muscles voire des manifestations rares à type de dextrocardie [3], agénésie rénale, pneumothorax du côté affecté [4], luxation de l'épaule, anomalie du bilan hémostatique et la thrombocytopénie, déficit en hormone de croissance [7] ou paralysie du nerf facial [10]. Dans cette observation, nous rapportons le cas d'une patiente répondant aux critères cliniques du syndrome de Poland, confirmé par un examen tomodensitométrique, qui est l'examen de choix pouvant révéler toutes les anomalies décrites au cours de ce syndrome [5, 7]; Il permet d'évaluer l'hypoplasie musculaire, en particulier pour le latissimus dorsi, mais aussi les anomalies osseuses associées de la cage thoracique (pectus excavatum ou carinatum associé) de façon plus précise que la radiographie thoracique. La TDM fournit alors une aide supplémentaire aux cliniciens à poser le diagnostic plus facilement et aux chirurgiens plasticiens pour être au courant des anomalies exactes et procéder à la reconstruction chirurgicale. La réalisation d'une IRM a été discutée par certains auteurs afin de réaliser un bilan précis des atteintes musculaires [5]. La mammographie est de réalisation systématique chez la femme adulte après 30 ans vu que l'association syndrome de Poland et atteinte néoplasique mammaire a été largement discutée [11]. Dans ce cas rapporté, nous n'avons noté aucune malformation associée des membres supérieurs, qui ne se produisent que dans 12% des patients atteints du syndrome de Poland. Mais des cas bénins du syndrome de Poland sans participation du membre supérieur ne peuvent pas être évidents jusqu'à la puberté, lorsque les différences entre les deux côtés deviennent plus apparentes [1]. La maladie est à l'origine d'altération de l'image corporelle du patient notamment chez les patientes de sex féminin avec le risque d'agénésie mammaire associée [7]. Selon la littérature, le syndrome de la Poland a de nombreuses présentations cliniques. Il pourrait être de diagnostic difficile pour les cliniciens et les chirurgiens plasticiens [7]. Une classification clinique et radiologique des anomalies musculosquelettiques thoraciques du syndrome est proposée [7], le classant en quatre catégories Tableau 1. Le traitement de ces anomalies repose sur la reconstruction chirurgicale qui devrait se faire dès l'âge de 13-14 ans chez les patients de sexe masculin ayant un latissimus dorsi intact [7]. Ce dernier est généralement récolté à travers une petite incision dorsale et axillaire et transposé pour combler le vide du à l'absence du muscle pectoralis major. La reconstruction par prothèse a été décrits pour les hommes, mais les résultats sont généralement décevants [12]. Chez les femmes, le développement anormal de la glande mammaire peut être à l'origine de préjudice esthétique et par conséquent psychologique, un traitement précoce peut être alors envisagé, une fois le développement des seins a été achevée [7].

**Tableau 1:** Classification radio-clinique du syndrome de Poland

Classification	Présentation clinique
1 er degré	Hypoplasie du muscle pectoral
2^ème^ degré	Absence de la portion sterno-costale du muscle pectoralis major
3^ème^ degré	Absence totale du pectoralis major ou des deux muscles pectoraux
4^ème^ degré	Hypoplasie ou absence des muscles pectoraux avec anomalies squelettiques du thorax (sternum ou cotes)

## Conclusion

Le syndrome de Poland est une malformation congénitale rare, associant à des degrés divers, malformations thoraciques et malformations du membre supérieur homolatéral. Le diagnostic est suspecté cliniquement. L'imagerie et essentiellement la TDM permet de confirmer le diagnostic de ce syndrome, d'en faire le bilan lésionnel complet et de dresser la stratégie thérapeutique appropriée à chaque forme. Ce traitement doit s'envisager également en fonction du sexe, de l'âge et de la sévérité de l'atteinte.

## References

[1] Frioui S, Khachnaoui F (2015). Poland's syndrome. Pan African Medical Journal..

[2] Friedman T, Reed M, Elliott AM (2009). The carpal bones in Poland syndrome. Skeletal Radiol..

[3] Iyer R, Parisi M (2012). Multimodality imaging of poland syndrome with dextrocardia and limb anomalies. Clinical Nuclear Medicine..

[4] Assadi FK, Salem M (2002). Poland syndrome associated with renal agenesis. Pediatr Nephrol..

[5] Chichery A, Jalbert F, Foucras L, Grolleau J-L, Chavoin J-P (2006). Syndrome de Poland. EMC, Techniques chirurgicales-Chirurgie plastique reconstructrice et esthétique..

[6] Sparks D, Adams B, Wagels M (2015). Poland's syndrome : an alternative to the “vascular hypothesis”. Surg Radiol Anat..

[7] Kapetanakis S, Papadopoulos C, Triantafilidis A, Fiska A, Agrogiannis N, Maria D, Panagiotou P (2012). Muscle abnormalities of the chest in Poland's syndrome: variations and proposal for a classification. Surg Radiol Anat..

[8] Legbo JN (2006). Poland's syndrome: report of a variant. J Natl Med Assoc..

[9] Chowdhury K, Chakrabortty R, Gope S (2015). Poland's syndrome: a case report and review of literature. J Pak Med Assoc..

[10] Gupta RK, Gupta RC, Deedar S (2003). An unusual presentation of Poland's syndrome. JK science..

[11] Cherradi Lachhab I, Dafiri R (2014). Syndrome de Poland et cancer mammaire controlatéral : une association exceptionnelle. EMC Imagerie de la Femme..

[12] Foucras L, Grolleau JL, Chavoin JP (2005). Poland's syndrome and hand's malformations: about a clinic series of 37 patients. Ann Chir Plast Esthet..

